# Long-term Memory Testing in Children With Typical Development and Neurodevelopmental Disorders: Remote Web-based Image Task Feasibility Study

**DOI:** 10.2196/39720

**Published:** 2023-05-08

**Authors:** Truong An Bui, Cory Scott Rosenfelt, Kerri Hope Whitlock, Mickael Leclercq, Savannah Weber, Arnaud Droit, Sandra A Wiebe, Jacqueline Pei, Francois V Bolduc

**Affiliations:** 1 Department of Pediatrics University of Alberta Edmonton, AB Canada; 2 Computational Biology Laboratory Centre de Recherche du CHU de Québec Université Laval Research Centre Québec City, QC Canada; 3 Department of Psychology University of Alberta Edmonton, AB Canada; 4 Neuroscience and Mental Health Institute University of Alberta Edmonton, AB Canada; 5 Department of Educational Psychology University of Alberta Edmonton, AB Canada; 6 Women and Children Health Research Institute University of Alberta Edmonton, AB Canada

**Keywords:** memory, neurodevelopmental disorder, autism spectrum disorder, intellectual disability, developmental delay, hippocampus, recognition, paired association learning, remote testing, autism, disorder, genetics, developmental, developmental disorder, game, remote, testing, diagnose, diagnosis

## Abstract

**Background:**

Neurodevelopmental disorders (NDD) cause individuals to have difficulty in learning facts, procedures, or social skills. NDD has been linked to several genes, and several animal models have been used to identify potential therapeutic candidates based on specific learning paradigms for long-term and associative memory. In individuals with NDD, however, such testing has not been used so far, resulting in a gap in translating preclinical results to clinical practice.

**Objective:**

We aim to assess if individuals with NDD could be tested for paired association learning and long-term memory deficit, as shown in previous animal models.

**Methods:**

We developed an image-based paired association task, which can be performed at different time points using remote web-based testing, and evaluated its feasibility in children with typical development (TD), as well as NDD. We included 2 tasks: object recognition as a simpler task and paired association. Learning was tested immediately after training and also the next day for long-term memory.

**Results:**

We found that children aged 5-14 years with TD (n=128) and with NDD of different types (n=57) could complete testing using the Memory Game. Children with NDD showed deficits in both recognition and paired association tasks on the first day of learning, in both 5-9–year old (*P*<.001 and *P*=.01, respectively) and 10-14–year old groups (*P*=.001 and *P*<.001, respectively). The reaction times to stimuli showed no significant difference between individuals with TD or NDD. Children with NDD exhibited a faster 24-hour memory decay for the recognition task than those with TD in the 5-9–year old group. This trend is reversed for the paired association task. Interestingly, we found that children with NDD had their retention for recognition improved and matched with typically developing individuals by 10-14 years of age. The NDD group also showed improved retention deficits in the paired association task at 10-14 years of age compared to the TD group.

**Conclusions:**

We showed that web-based learning testing using simple picture association is feasible for children with TD, as well as with NDD. We showed how web-based testing allows us to train children to learn the association between pictures, as shown in immediate test results and those completed 1 day after. This is important as many models for learning deficits in NDD target both short- and long-term memory for therapeutic intervention. We also demonstrated that despite potential confounding factors, such as self-reported diagnosis bias, technical issues, and varied participation, the Memory Game shows significant differences between typically developing children and those with NDD. Future experiments will leverage this potential of web-based testing for larger cohorts and cross-validation with other clinical or preclinical cognitive tasks.

## Introduction

### Neurodevelopmental Disorders Are Common

Learning new information by forming associations is at the core of development and daily functioning. Yet, our understanding of how such associations may differ between typically developing individuals and those with neurodevelopmental disorders (NDD) remains insufficient in some important aspects. NDD includes a group of diagnoses, in which the development of typical brain functions, such as attention, cognition, or social functioning, is altered [1]. Examples of common NDDs include attention-deficit/hyperactivity disorder (ADHD), autism spectrum disorder (ASD), and intellectual disability [2]. In the United States alone, approximately 1 in 6 (17%) children between the ages of 3 and 17 years had an NDD, as reported by the parents [3]. This mirrors the rate identified in population studies worldwide [4-10]. Not only individuals with NDD but also their families will experience significant financial and psychological burdens [11-13], necessitating more effort in developing targeted interventions, as most NDD will have lifelong effects [14].

### Current Discrepancies in the Memory Type Tested Between Preclinical and Clinical NDD Models

In the last decade, there has been a vast increase in studies identifying genes associated with each neurodevelopmental condition and the development of multiple animal models to study the disorders [15-17]. Nevertheless, there is a disparity between the cognitive testing used in humans and the cognitive measures used in animal models, which may hamper the translation of candidate treatments [18,19].

As many of the genes discovered in NDD belong to signaling pathways that were already investigated in animal models of learning and memory [20,21], many well-established “memory assays” previously used in NDD animal models do not necessarily align with NDD clinical testing. Animal testing has been focusing on studying nonassociative (sensitization and habituation) versus associative memory (fear conditioning, spatial navigation, and olfactory conditioning) [22,23], and short-term memory (STM; training and recall within minutes) versus long-term memory (LTM; 24 hours) [24]. Mutants for NDD gene orthologues and candidate pharmacological interventions have been characterized for their effects on those behaviors [25-27]. The tools to detect memory problems of NDD individuals in clinical settings, however, remain inadequate or rely heavily on self-reports [28,29].

While some of the pioneering work on memory and recall in typical individuals, such as one by Ebinhaus, investigated STM and LTM [30], most of the clinical memory testing has been focusing on STM types, such as working memory [31]. The majority of individuals with NDD, including those with common genetically defined conditions such as Fragile X syndrome [32-35], Down syndrome [36,37], Williams syndrome [38], as well as clinically defined disorders, such as specific language impairment [39,40], ADHD [41-43], ASD [44], and intellectual disability [45-48], have been found to have STM defects. LTM, however, has not been reported, except in 1 study done in the 1990s, showing LTM defects in Down syndrome [49]. We hypothesize that this is partly due to technical and financial limitations that limit the feasibility of developing novel and highly accurate memory testing, and repeated testing in a lab setting. This suggests that web-based or remote testing might be the solution.

Similarly, limitations in the current test design have limited consistent use of memory testing in NDD. Paired association is often employed clinically with the use of picture and name or word-word association [50]. This type of test relies on hippocampal functioning [51-54]. Typically, an individual will learn an association between a person’s picture and their name. At testing time, they will be presented with the photo and 3 names (1 correct and 2 other names of different individuals seen in the training session). Those with NDD can have challenges in reading and have, therefore, not been exposed to paired association testing extensively [55,56]. Nevertheless, picture-based association tasks have been used for children with dyslexia [22,57] and NDD [58] to circumvent issues with language delay [59,60].

A potential issue in remote testing would be to infer the degree of attention of the participants. Control tasks [61], such as recognition of a pair, a less difficult, hippocampal-independent task, have been used to test the ability of an individual to participate and their attention to a task [54,62,63]. In this situation, using flanker images, which have never been seen before, makes recalling the association easier.

### Emergence of Web-based and Remote Testing as a Method for Cognitive Assessment in NDD

Touch screen-based testing has emerged as an accurate and engaging tool for testing children and individuals with NDD. Evidently, subtasks of the NIH Toolbox for the Assessment of Neurological and Behavioral Function, such as episodic memory [64] and working memory [65], have been used recently in a clinical trial for Fragile X syndrome [66]. However, those tasks do not assess associative or LTM skills. Additionally, the NIH toolbox currently needs to be administered locally on tablets, with guidance from trained researchers in a laboratory. Another test that is used for individuals with NDD is the Cambridge Neuropsychological Test Automated Battery, which evaluates working memory, episodic memory, attention, and decision-making [67]. This test is licensed and has been administered in individuals with ADHD using researchers’ tablets [68,69]. While being well constructed, these tests currently do not allow for the testing of associative memory at different time points. They are also limited to in situ testing in the laboratory or on researchers’ devices, making it expensive and inaccessible to a large number of participants. Testing in traditional laboratory environments can also be challenging for children, especially those with NDD, considering the high prevalence of anxiety [70-76].

Thus, we aim to develop a remote web-based and accessible cognitive test, which could better mirror the type of memory (associative) and the different time points (STM and LTM) used in preclinical models, in hope that this will make preclinical therapies more easily translated into clinical trials. Here, we assessed the feasibility of such testing administered web-based in individuals with typical development (TD) and NDD.

## Methods

### Ethics Approval

The project was approved by the ethics committee at the University of Alberta (Pro00033138).

### Informed Consent

**Figure 1:**
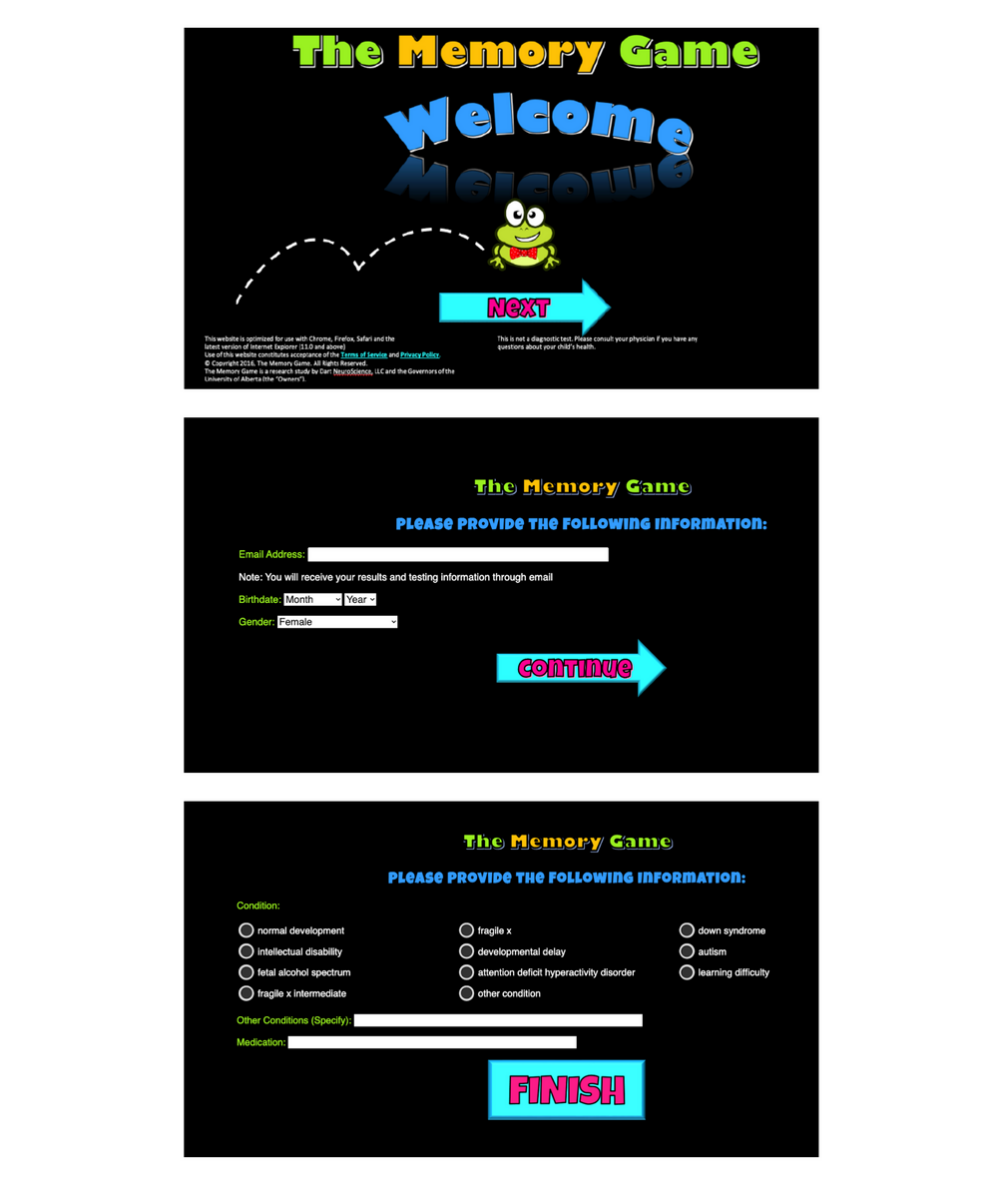
Registration. Participants or caregivers enter their email addresses (to receive the participant code) and then provide demographic information as well as the diagnosis they have (if applicable).

Informed consent to participate in the study was sought from the participant’s parent or guardian (Multimedia Appendix 1). Next, participants, or parents and caregivers, were asked to provide information about the participant’s age, neurological and medical conditions, sex, and current medications (Figure 1).

### Participants

In total, 128 individuals with no known neurological conditions and 57 individuals with NDD, including developmental delay, ASD, and intellectual disability participated in the study. Eligible participants’ caregivers self-reported their diagnoses or the absence thereof. Table 1 includes the demographic information for the participants. The participants were contacted by their School Board via the Healthy Infants and Children Clinical Research Program (HICCUP) or via family support groups. The access was free of charge to participants. The participants received the URL for the Memory Game.

**Table 1:** Demographic characteristics of participants.

Condition and age group		Day 1, n	Day 2, n
**Typical development**			
	5-9 years	97 (female: 4, male: 4, not available: 89)	56 (female: 4, male: 2, not available: 50)
	10-14 years	31 (female: 3, male: 3, not available: 25)	17 (female: 0, male: 2, not available: 15)
**Neurodevelopmental disorders**			
	5-9 years	30 (female: 7, male: 6, not available: 17)	16 (female: 5, male: 4, not available: 7)
	10-14 years	27 (female: 3, male: 6, not available: 18)	19 (female: 3, male: 6, not available: 10)

### The Memory Game Interface

All participation in the Memory Game was done web-based using a touch screen tablet device (eg, iPad). The procedure has five main components: (1) consent, (2) registration, (3) tutorial video, (4) training phase, and (5) testing phase.

**Figure 2:**
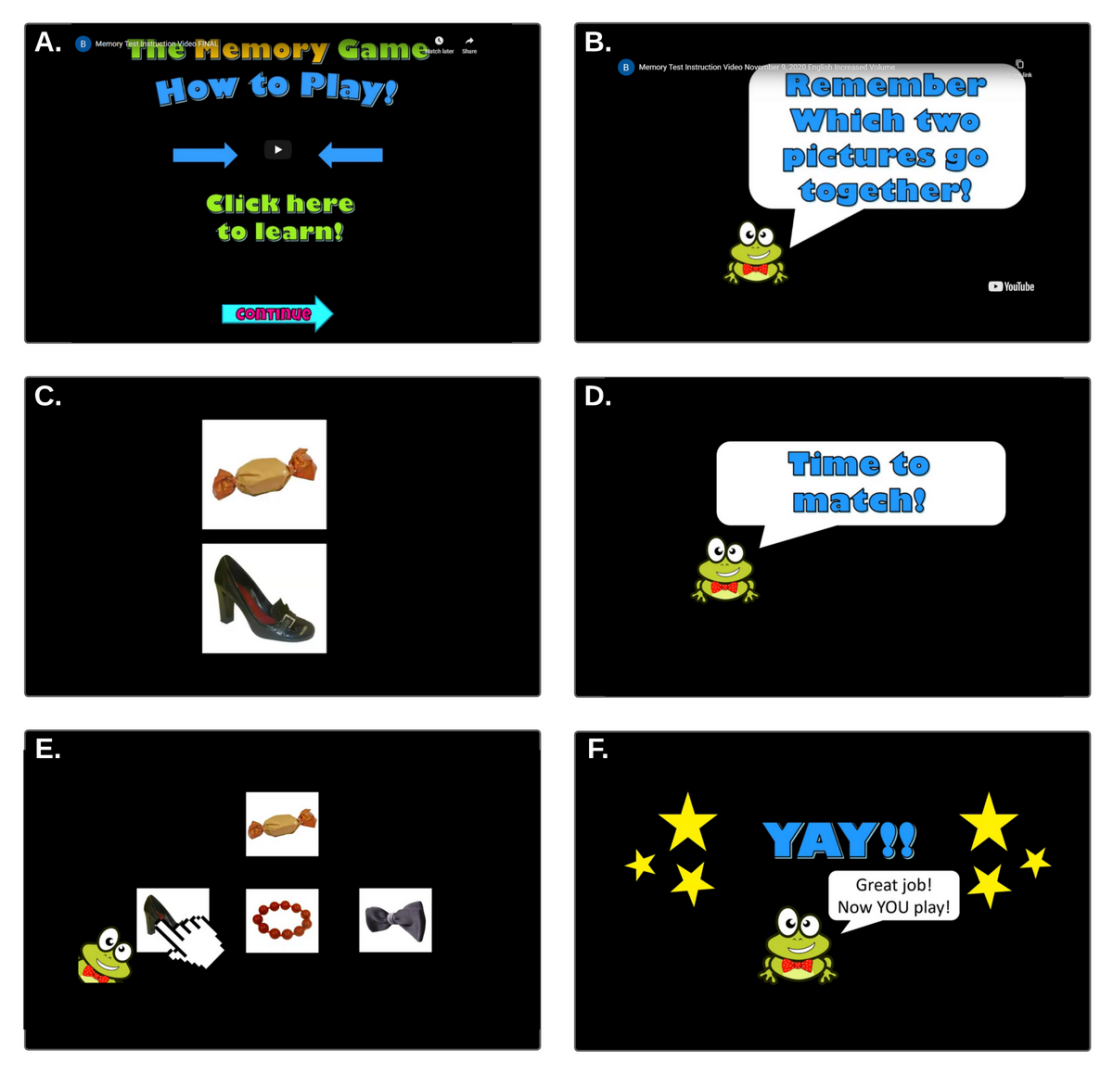
Tutorial video demonstration. In order to explain the task as easily as possible (without direct supervision from the researchers), a tutorial video explaining the procedure to the participants was developed. This includes a voiceover reinforcing the information provided visually about the pictures that go together.

Having completed the consent and registration, participants received a code via email, which allows them to move on to the training and testing components. These occur over 2 days. On day 1, the participants participate in 3 phases of the game: a tutorial phase, a practice phase, and a testing phase. The phases had to be completed to proceed through the test. The tutorial phase consisted of verbal and visual explanations of completing the test by properly matching the pairs. This is in the form of an instructional video to explain how the game works. A cartoon frog demonstrates with examples, while a voiceover explains what to do (Figure 2). The video is followed by the practice phase, in which participants are first shown 6 sets of pairs, followed by 6 questions. In this section, they were given feedback as to whether their response was correct or incorrect. Finally, participants went through the testing phase where they were shown 20 sets of pairs followed by 20 questions, this time without any feedback on their responses. On day 2, the participants were directed to the testing phase (as on day 1) and were not given feedback on their responses.

### Task Description

**Figure 3:**
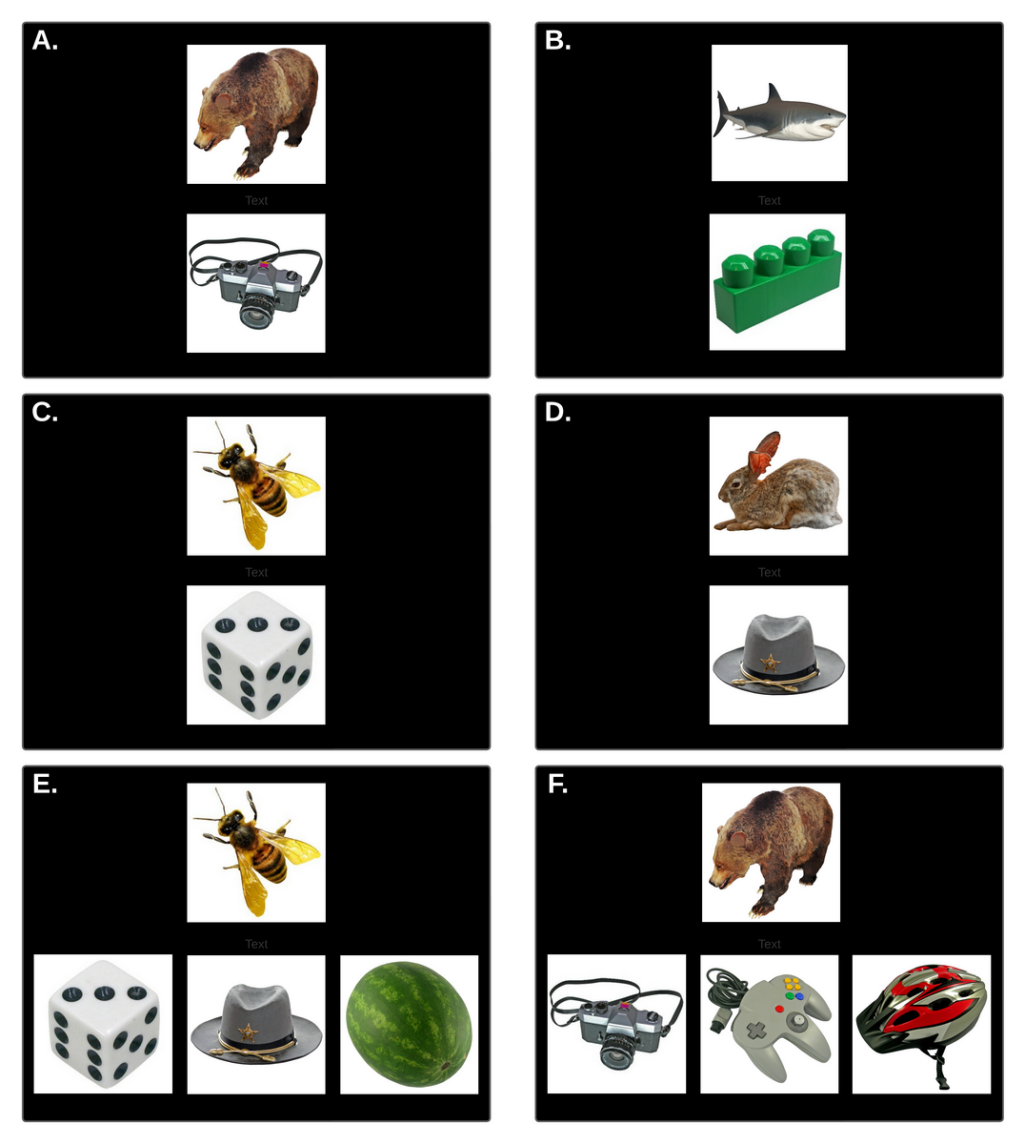
Example of stimuli and paradigms used in the Memory Game. (A-D) Representative examples of pairs of images used in the training phase. The testing phase consists of 2 different types of tasks: paired association (PA) and recognition (R). (E) In the PA task, the participant must distinguish the correct association (the bee goes with the dice as seen in training 3C) but with flanker images present that were part of other associations presented in the training phase (hat from training 3D), making the task more difficult. (F) For the R task, the target image from the pair (the camera which goes with the bear) is flanked by 2 pictures, which were not seen in the training phase.

The Memory Game includes questions testing both paired association and recognition memory, depending on the distractor photos present (Figure 3). In a recognition question, the 2 distractor photos are not part of any pairs seen in training and have not been previously viewed. In a paired association question, 1 of the distractors will be the matching photo for a prompt not currently being displayed and so will have been previously viewed. The images used have been selected based on their shape and appearance from the Bank of Standardized stimuli (BOSS) database created by Dr Mathieu Brodeur.

### Task Development

We initially developed the Memory Game for testing using E-Prime (Psychology Software Tools) and administered it to children with NDD in our developmental neurology clinic, using a touch screen laptop under the supervision of trained undergraduate neuroscience students. This allowed us to optimize the instruction video and the choice of images, and refine the task. For instance, we found that having a voiceover during the instruction video made it easier for the participants and caregivers to follow. The choice of the pictures used for the paired association and recognition testing, which were standardized in the BOSS database, was reviewed for general understandability and quality assurance with caregivers and for formatting (orientation, color, and shapes) with 2 psychologists from our team (SAW and JP). Similarly, we changed the position of the “correct response” from left, middle, and right to prevent a “false success” if, for instance, an individual always chose a given position. We also sought feedback from caregivers present in the room during the testing about the flow used and challenges in understandability. In addition to general comments, we ensured that the participants understood the instruction video and the training and testing phases of the Memory Game. Once established in E-Prime software, a widely used program for stimulus presentation [77], the final version of the task was discussed with the programmers (Jeffrey Van Alstine, Arthur Schuiltz, Department of Education from University of Alberta) and developed for web-based testing on tablets. The Memory Game underwent several revisions to optimize responsiveness. While diagnosis and age were included in all versions, sex was added in the second version only.

### Coding Approach

The Memory Game consists of 3 parts: the front-end game interface, the researcher interface, and the application programming interface (API) that both communicate with. The front-end game interface is static HTML running a custom web application built with jQuery 1.11 (OpenJS Foundation) and reliant on the YouTube iFrame API. The researcher interface is a small custom PHP 7 application with no dependencies. The API itself is constructed on the PHP Slim framework v3.4, uses PHPMailer v5.3 to send confirmation or reminder emails through a local university SMTP server, and in turn saves the data in a MySQL (Oracle Corporation) database with any identifying information (email addresses) encrypted such that it can only be decoded and read by researchers with the correct key. The Memory Game is web based and can be accessed in [78].

### Data Analysis

From the game component, the percentage of correct responses was recorded, as well as the time required to answer each question as reaction time (RT).

The performance of participants with NDD was compared against the corresponding age group of the TD participants. The groups were broken down into 2 age groups: 5-9 years and 10-14 years. The percent correct score is given as a percentage of questions answered correctly. The performance on day 1 and day 2 was plotted. A line joining those 2 was drawn and the slope was used to estimate memory decay. RT is the average time participants took to answer the questions and is given in ms. Unpaired *t* tests with assumed Gaussian distribution (using parametric test), no assumption about consistent SDs (the Welch *t* test), and no correction for multiple comparisons were performed (*P* value * <.05, ** <.01, *** <.001; Cronbach α=.05). Effect sizes between groups were reported as Cohen *d* values. All statistical analyses were run using Prism software (GraphPad).

### Outlier Analysis

For RT, any outliers for a participant were detected using a modified Z-score [79], adapted for small data sets, and then removed. This was done on a participant-by-participant basis, with any absolute value greater than 3.5 labeled as an outlier.

## Results

### Paired Association and Recognition Performance on Day 1

We recruited individuals with TD and individuals with different types of NDD. These were self-reported by the users’ parents and included developmental delay, intellectual disability, and ASD (Table 2).

We started by assessing learning performance (day 1) for individuals with NDD compared to individuals with TD. We evaluated both performance and RT for each type of paradigm (paired association and recognition) in the function of age group (5-9 years old and 10-14 years old).

The typical session length ranged from 3 to 6 minutes. Not all participants completed the test on day 1 after initiating it. For day 1, 69 TD and 32 NDD individuals started but did not finish the practice portion, while 32 TD and 14 NDD individuals finished the practice but not the testing portion. For day 2, among participants that completed day 1, 5 TD and 12 NDD started but did not finish the testing.

**Figure 4:**
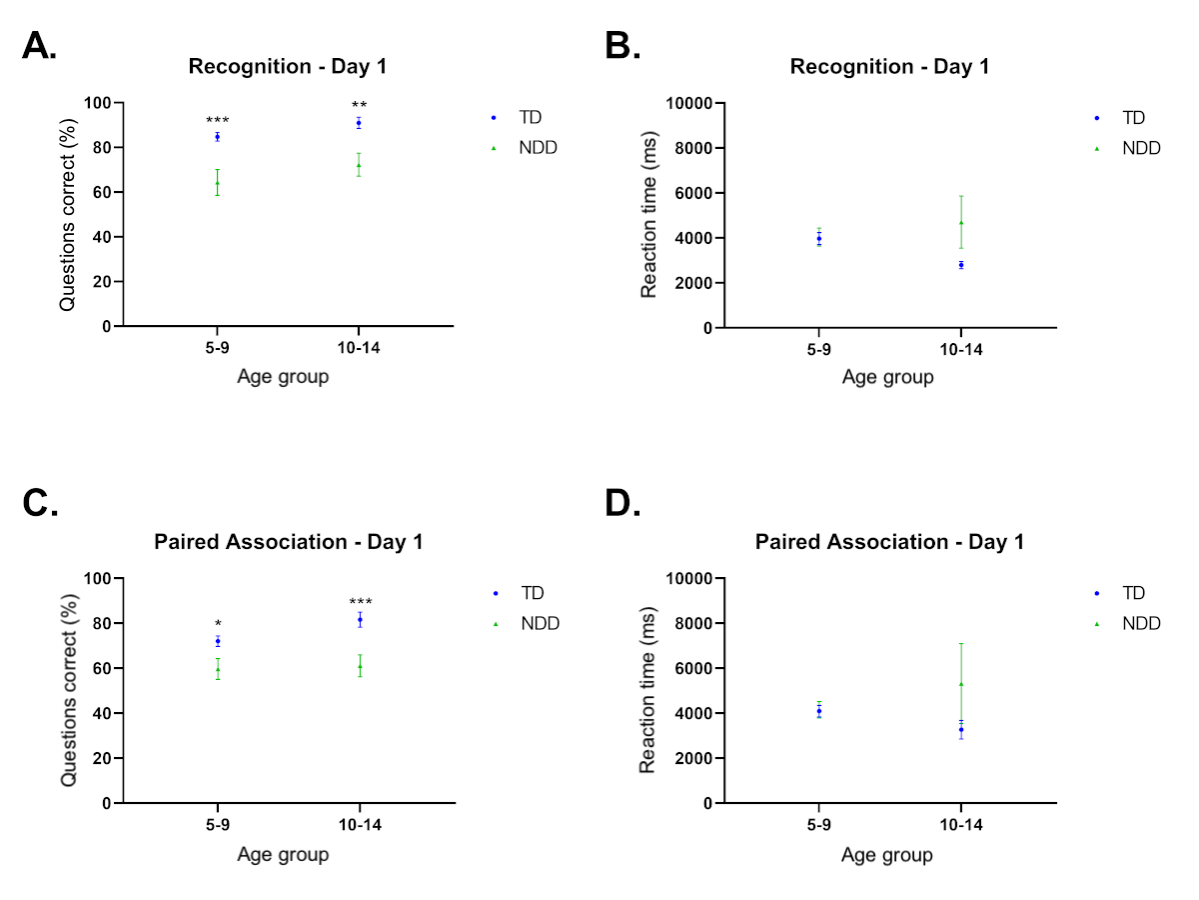
Performance in recognition (R) and paired association (PA) tasks. (A) Performance of individuals with typical development (TD) and neurodevelopmental disorders (NDD) in the recognition task by age group. (B) Reaction time (RT) in the R task in individuals with TD and NDD by age group. (C) Performance for the PA task in individuals with TD and NDD by age group. (D) RT for the PA task in individuals with TD and NDD by age group. TD ages 5-9 years, day 1: n=97; TD ages 10-14 years, day 1: n=31; NDD ages 5-9 years, day 1: n=30; NDD ages 10-14 years, day 1: n=27. *t* tests were performed to assess differences. *P<.05, **P<.01, ***P<.001.

NDD performance was significantly lower for recognition memory in both age groups (ages 5-9 years, *P*<.001, *d*=0.89; ages 10-14 years, *P*=.001, *d*=0.90) (Figure 4A). We found that RT was not significantly different between TD and NDD individuals (ages 5-9 years, *P*=.90; ages 10-14 years, *P*=.09) (Figure 4B). We observed a trend toward decreased RT from 5-9 to 10-14 years in TD, but we did not observe such a reduction in NDD individuals.

**Table 2:** Distribution of diagnosis by age and day of memory test performance.

Condition	Day 1		Day 2	
	Ages 5-9 years (n=30), n	Ages 10-14 years (n=27), n	Ages 5-9 years (n=16), n	Ages 10-14 years (n=19), n
Autism	7	3	4	2
Developmental delay	15	12	9	9
Intellectual disability	0	1	0	1
Multiple conditions	8	11	3	7

For paired association memory, we found significant deficits in 5-9 years old children (*P*=.01, *d*=0.53) and 10-14 years old (*P*<.001, *d*=0.94) with NDD compared to TD (Figure 4C). RT did not show significant differences (ages 5-9 years, *P*=.91; ages 10-14 years, *P*=.23) but showed a trend for decreased RT in TD (Figure 4D) between the 2 age groups. RT in NDD trended toward increasing when comparing 5-9 years old to 10-14 years old individuals, suggesting that NDD performance potentially requires more cognitive processing, and thus the increased reaction time.

We also found that recognition scores were higher than the paired association. This difference was observed for both groups; however, it was only significant for the TD groups (ages 5-9 years, *P*<.001, *d*=0.60; ages 10-14 years, *P*=.03, *d*=0.57) and not the NDD groups (ages 5-9 years, *P*=.53; ages 10-14 years, *P*=.12).

### Memory Decay From Day 1 to Day 2

**Figure 5:**
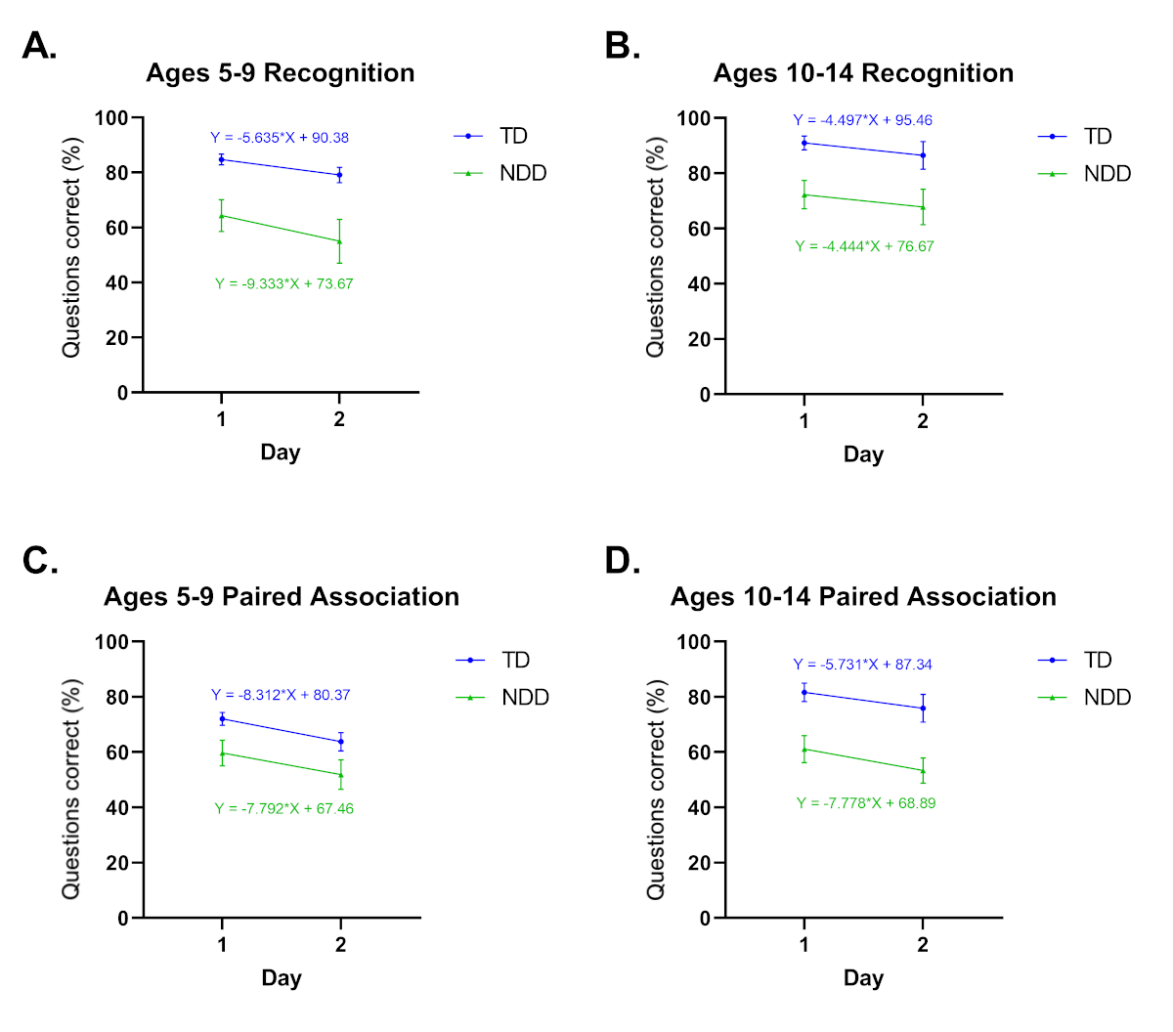
Comparative performance right after training (day 1) compared to performance the day after (day 2). (A) Performance of individuals with typical development (TD) and neurodevelopmental disorders (NDD) in the recognition task by age group. (B) Performance in the recognition (R) task in individuals with TD and NDD by age group. (C) Performance for the paired association (PA) task in individuals with TD and NDD by age group. (D) Performance for the PA task in individuals with TD and NDD by age group. TD ages 5-9 years, day 1: n=97; TD ages 5-9 years, day 2: n=56; TD ages 10-14 years, day 1: n=31; TD ages 10-14 years, day 2: n=17; NDD ages 5-9 years, day 1: n=30; NDD ages 5-9 years, day 2: n=16; NDD ages 10-14 years, day 1: n=27; NDD ages 10-14 years, day 2: n=19.

We found that for the recognition task, 5-9 years old individuals with NDD presented with a faster decay in performance on day 2 (24-hour decay) compared to individuals with TD, as shown by the difference in slope (Y=5.637 in TD vs Y=9.333 in NDD) (Figure 5A). Nevertheless, this was not observed in individuals aged 10-14 years (Figure 5B). Strikingly, the opposite trend was observed for the paired association. Individuals with NDD showed similar decay in the 5-9 years old group (Figure 5C) but had higher decay in 10-14 years old (Figure 5D). No differences were observed in any of the reaction time comparisons (Multimedia Appendix 2). There was similar attrition in participation from day 1 to day 2 at all ages for both TD and NDD groups (41 out of 97 participants [42%] for individuals with TD vs 14 out of 30 [47%] for those with NDD in the 5-9 years old group, and 14 out of 31 participants [45%] in TD vs 12 out of 27 (44%) in individuals with NDD in the 10-14 years old group).

### Performance Distribution Homogeneity Between Groups

**Figure 6:**
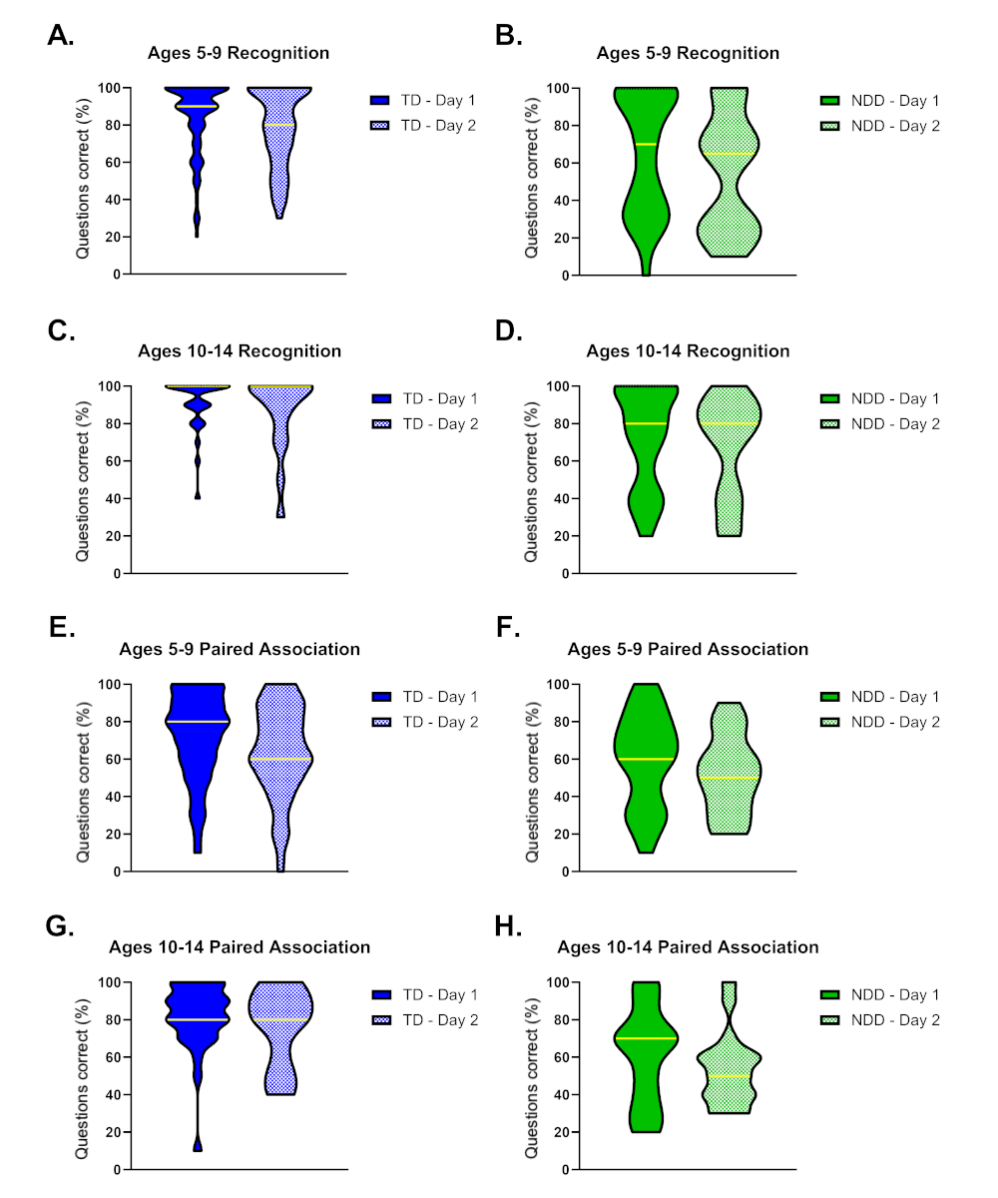
Distribution of performance on day 1 and day 2 for individuals with typical development (TD) and neurodevelopmental disorders (NDD). (A) Memory performance for recognition (R) in individuals with TD of the 5-9 years old group. (B) Memory performance for R in individuals with NDD of the 5-9 years old group. (C) Memory performance for R for individuals with TD of the 10-14 years old group. (D) Memory performance for R for those with NDD of the 10-14 years old group. (E) Memory performance for paired association (PA) for individuals with TD of the 5-9 years old group. (F) Memory performance for PA for individuals with NDD of the 5-9 years old group. (G) Memory performance for PA for individuals with TD of the 10-14 years old group. (H) Memory performance for PA for individuals with NDD of the 10-14 years old group. TD ages 5-9 years, day 1: n=97; TD ages 5-9 years, day 2: n=56; TD ages 10-14 years, day 1: n=31, TD ages 10-14 years, day 2: n=17; NDD ages 5-9 years, day 1: n=30; NDD ages 5-9 years, day 2: n=16; NDD ages 10-14 years, day 1: n=7; NDD ages 10-14 years, day 2: n=19.

We compared individuals from TD and NDD by plotting their performance and RT in both paired association and recognition to demonstrate homogeneity. Several individuals with NDD had mixed diagnoses; hence, we could not plot them by diagnosis individually. We found that those with NDD appeared to have a different group distribution in performance, especially in the younger age group (5-9 years old), with a bimodal distribution for recognition and paired association (Figure 6). On the other hand, RT had a more uniform distribution (Multimedia Appendix 3).

## Discussion

### Feasibility of Remote Web-based Cognitive Testing

Our work shows that remote web-based memory testing is feasible for children with TD (n=128 individuals) and NDD (n=57 individuals), as early as 5 years old. Indeed, using picture matching allowed for the testing of children with NDD. The results are also consistent with a previous report of a progressive increase in performance for recognition tasks in children with TD [80]. Our task also allowed us to measure paired association memory and find significant differences between participants with TD versus NDD.

It also found that children with TD or NDD were able to perform LTM (day 2) testing in the same proportion, supporting further the feasibility of the Memory Game as a tool to probe both STM and LTM. In addition, a significantly decreased performance in individuals with NDD compared to TD was found.

### Benefits of Web-based Testing

We believe that the tablet approach makes cognitive testing more engaging for children. Evidently, we observed during our initial pilot testing (done in person) that most children enjoyed the game. In addition, by using pictures, we could help test individuals with limited literacy and language development, who could not perform traditional word-matching tasks. The task could be extended also to individuals using other languages; however, this will require updating the instruction video. Considering the prevalence of anxiety in individuals with NDD [67,81], we propose that remote testing with the Memory Game, being complete in the individual’s familiar environment, may better capture the full potential of individuals with NDD.

Web-based testing on participants’ devices is important as remote testing has been increasingly considered by researchers to resolve problems with participant compensation for travel, geographical issues in rare disorders, and recruiting large numbers of participants.

While this work focuses on clinical diagnosis (NDD), having access to a larger number of participants will be key in future work focused on specific genes or syndromes. In addition, the closer proximity of the cognitive measures tested by the Memory Game (LTM and paired association) could facilitate the translation of findings from animal models.

### Current Limitations

One of the trade-offs of remote web-based testing is in ascertaining the behavior of the participant as one would do with in-person testing. For instance, an individual may be distracted and perform suboptimally. The participant and caregiver may also not understand the instruction video, and thus may not be able to complete the task optimally. While we aimed to develop the instruction video with the feedback of caregivers during the early pilot testing in the clinic, it is possible that some users may not have understood it well. It is also challenging to assess how much and what type of guidance was provided by caregivers. Another important technical improvement to consider would be the flow of the test so that the next step is dependent and determined by the performance of the participant in the practice trial. At the moment, even though the participant receives feedback, they can still proceed to the testing phase regardless of their previous performance. It may be important to redemonstrate to the participant the tutorial or to train them further before they could proceed to the testing phase.

Some aspects of NDD such as repetitive behavior, or motor delay, may also impact the performance and thus, not reflect their memory capacity accurately. In addition, visual impairment could also influence the ability of participants to recognize the pictures and should be included in the future version as an item that would be reported by caregivers. Web-based testing also relies on self-reported diagnosis by caregivers, which might be incorrect. Therefore, future testing will include a correlation with in-person psychometric testing. While testing in larger cohorts may allow limiting the proportion of such outliers, smaller cohorts may be more impacted by such types of limitations. We also proposed an outlier analysis to identify individuals who may have experienced technical difficulties or stopped the test due to lack of attention or compliance, so as not to bias the results. Future research will be needed to develop adjustments for technical aspects, such as internet speed, device types, and browsers, which could also affect the results. Finally, one could also record the individual while performing the task. This has been implemented widely, for instance, during COVID-19 for web-based test administration in universities, but this presents many privacy issues and therefore was not used in the Memory Game.

In addition, despite the Memory Game allowing extensive testing by removing potential physical or geographical barriers, it remains challenging to achieve diversity of age, sex, gender, and diagnosis. Moreover, despite being potentially easier by being image-based rather than text-based paired association tasks, we recognize that many individuals with NDD may still not be able to perform the testing. In order to be of extensive use, the tutorial video will need to be translated, as well as some of the buttons, but the pictures used will be readily reusable.

This study also measured performance across different age groups but does not provide longitudinal data about memory performance in function of previous performance. Therefore, longitudinal follow-up will be important. We also recognize the importance of including individuals with diverse NDD etiology or a larger sample size of individuals, with a given specific NDD diagnosis to further assess diversity in the future and correlate the Memory Game findings with other cognitive tests such as for attention, working memory, intelligence quotient, or simpler form of plasticity (sensitization and habituation).

Future research will be needed to correlate the Memory Game performance with specific diagnoses and identify if the genes involved more specifically in LTM in animal models would affect LTM more than STM. In addition, repeated training has been shown to be important for LTM in typically developing individuals, for instance, when learning a language [82,83]. It will be interesting to evaluate if individuals with NDD would perform similarly on such tasks; however, we did not include those paradigms at this time based on the feedback of families in our initial development phase mentioning the importance of keeping the task short.

## Appendix

Multimedia Appendix 1 Online consent. The participants (or caregivers) first see the landing page with the Memory Game logo and are then directed to a consent form (approved by the University of Alberta). The participant (or caregiver) can then decide whether to give consent or not.

Multimedia Appendix 2 Comparative reaction time right after training (day 1) compared to performance the day after (day 2). (A) Reaction time (RT) of individuals with typical development (TD) and neurodevelopmental disorders (NDD) in the recognition (R) task by age group. (B) RT in the R task in individuals with TD and NDD by age group. (C) RT for the paired association (PA) task in individuals with TD and NDD by age group. (D) RT for the PA task in individuals with TD and NDD by age group. TD ages 5-9 years, day 1: n=97; TD ages 5-9 years, day 2: n=56; TD ages 10-14 years, day 1: n=31; TD ages 10-14 years, day 2: n=17; NDD ages 5-9 years, day 1: n=30; NDD ages 5-9 years, day 2: n=16; NDD ages 10-14 years, day 1: n=27; NDD ages 10-14 years, day 2: n=19.

Multimedia Appendix 3 Distribution of reaction time on day 1 and day 2 for individuals with typical development (TD) and neurodevelopmental disorders (NDD). (A) Reaction time (RT) for recognition (R) of individuals with TD of the 5-9 years old group. (B) RT for recognition of individuals with NDD of the 5-9 years old group. (C) RT for R for individuals with typical development of the 10-14 years old group. (D) RT for R for individuals with NDD of the 10-14 years old group. (E) RT for paired association (PA) for individuals with TD of the 5-9 years old group. (F) RT for PA for individuals with NDD of the 5-9 years old group. G) RT for PA for individuals with TD of the 10-14 years old group. H) RT for PA for individuals with NDD of the 10-14 years old group. TD ages 5-9 years, day 1: n=97; TD ages 5-9 years, day 2: n=56; TD ages 10-14 years, day 1: n=31; TD ages 10-14 years, day 2: n=17; NDD ages 5-9 years, day 1: n=30; NDD ages 5-9 years, day 2: n=16; NDD ages 10-14 years, day 1: n=27; NDD ages 10-14 years, day 2: n=19.

## Abbreviations

ADHD: attention-deficit/hyperactivity disorder
API: application programming interface
ASD: autism spectrum disorder
BOSS: Bank of Standardized stimuli
LTM: long-term memory
NDD: neurodevelopmental disorders
RT: reaction time
STM: short-term memory
TD: typical development
