# Supplementary figures and images for: Long-term Memory Testing in Children With Typical Development and Neurodevelopmental Disorders: Remote Web-based Image Task Feasibility Study
Source: JMIR Pediatr Parent. 2023 May 8;6:e39720. doi: 10.2196/39720 (PMC10203931; doi:10.2196/39720)

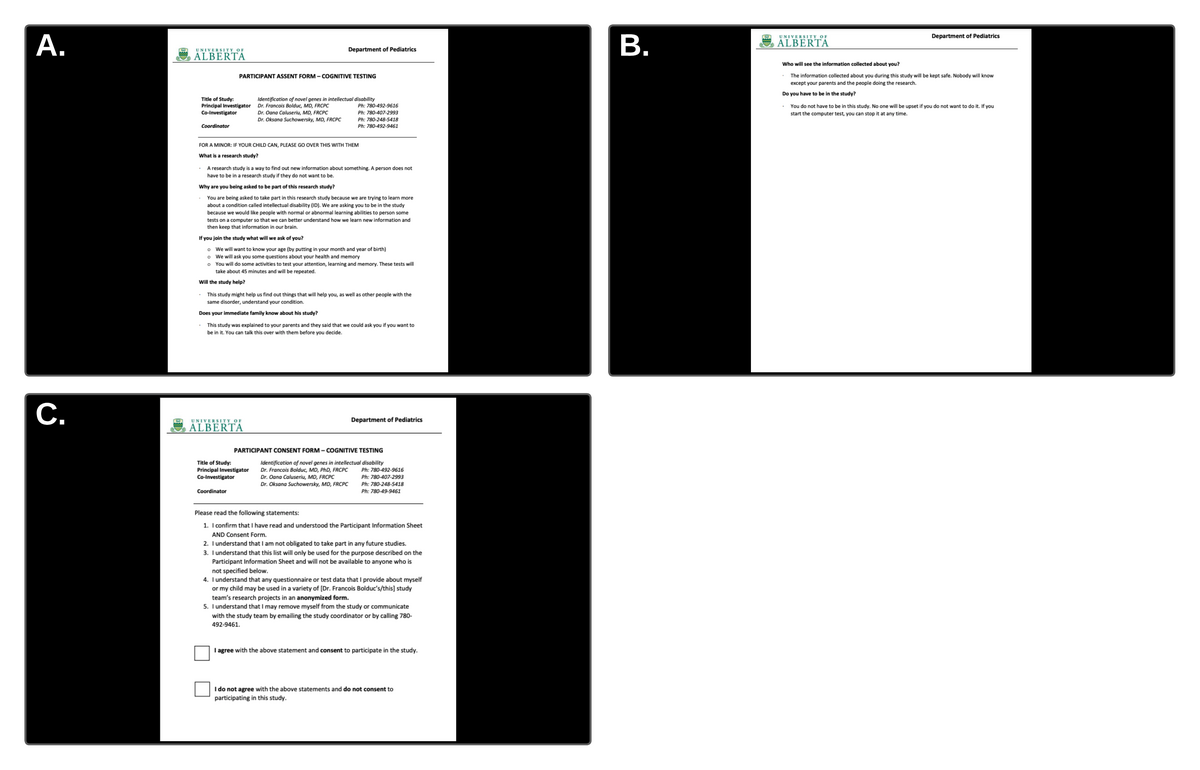

Supplement: Multimedia Appendix 1 [file pediatrics_v6i1e39720_app1.png]

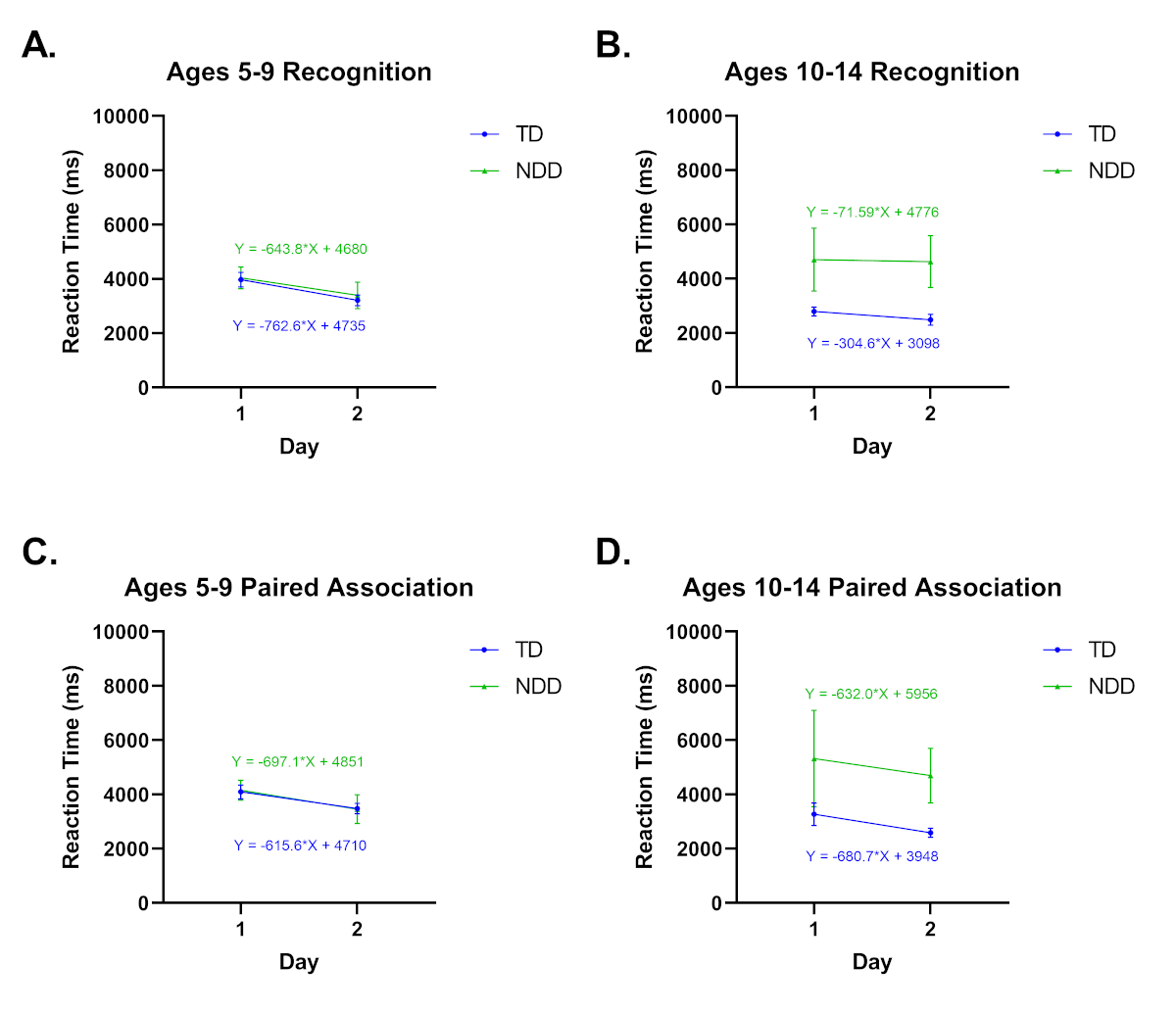

Supplement: Multimedia Appendix 2 [file pediatrics_v6i1e39720_app2.png]

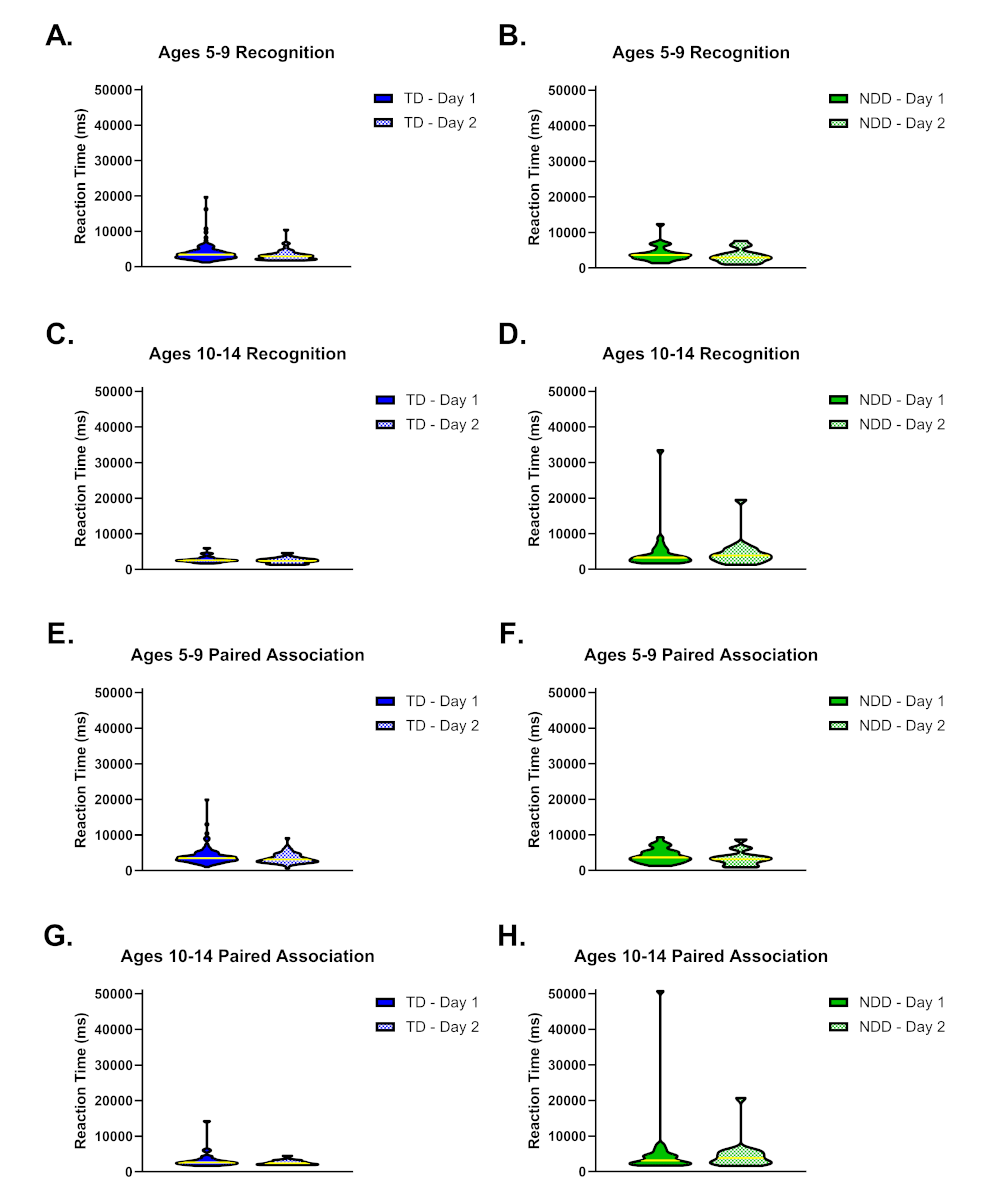

Supplement: Multimedia Appendix 3 [file pediatrics_v6i1e39720_app3.png]
